# Clinical Utility of Serum Cystatin C in Predicting Coronary Artery Disease in Patients Without Chronic Kidney Disease

**DOI:** 10.1002/jcla.21665

**Published:** 2014-01-29

**Authors:** Azza Dandana, Imen Gammoudi, Abdelkader Chalghoum, Hinda Chahed, Faouzi Addad, Salima Ferchichi, Abdelhedi Miled

**Affiliations:** ^1^ Department of Clinical Biochemistry CHU‐Farhat Hached Sousse Tunisia; ^2^ Department of Cardiology, CHU‐Fattouma Bourguiba Monastir Tunisia

**Keywords:** Estimated glomerular filtration rate, cystatin C, coronary artery disease, biochemical atherosclerosis markers

## Abstract

**Background:**

Cystatin C has been proposed as a novel marker of renal function and predictor of cardiovascular risk. The aim of this study was to investigate the role of cystatin C level as a predictor of cardiovascular events in patients with coronary artery disease (CAD).

**Methods:**

Three hundred and five coronary artery patients were included in this study. Serum cystatin C levels, high‐sensitive C‐reactive protein (hs‐CRP), and oxidative stress were measured. Estimated glomerular filtration rate (eGFR) and the CAD severity score were calculated.

**Results:**

Cystatin C was correlated with the CAD severity score (*r* = 0.631, *P* < 0.0001) and was significantly elevated in the CAD severity score >50. Every 0.1 mg/l increase in cystatin C, 2 mg/l increase in hs‐CRP, 0.2 mmol/l decrease in high‐density lipoprotein cholesterol, 13.7 ml/min decrease in eGFR, and 1.51 μmol/l increase in homocysteine caused a 34, 12, 5, and 22% increase in the risk of having CAD, respectively.

**Conclusion:**

Cystatin C could be a useful laboratory biochemical marker in predicting the severity of CAD. Cystatin C is associated with biochemical atherosclerosis markers such as CRP and homocysteine.

## INTRODUCTION

Renal function has been identified as a risk factor for the onset and prognosis of coronary artery disease (CAD, 1). Consequently, subjects with chronic renal failure are exposed to increased morbidity and mortality as a result of cardiovascular events. Further, accelerated cardiovascular disease (CVD) is a frequent complication of renal disease 2.

Glomerular filtration rate (GFR) is an important indicator of renal function. However, in practice, since GFR is usually not directly measured in routine clinical practice, markers such as creatinine are used to estimate GFR. Estimating equations such as the Cochroft‐Gault equation 3 or the modification of diet in renal disease (MDRD, 4) study equation are widely used for this purpose.

In recent years, cystatin C has been proposed as a more reliable marker of renal function than serum creatinine, in particular for the detection of small reductions in GFR 5. Cystatin C is a 122 amino acid, 13‐kDa protein that is a member of the family of competitive inhibitors of lysosomal cystein protease. It is encoded by the housekeeping type CST3 gene, and produced by all nucleated cells at a constant rate. The protein is located extracellularly and detected mainly in biological fluids. Because of its smaller size and cationic nature, it is freely filtered by the glomerulus. It is not secreted, but reabsorbed by tubular cells and subsequently catabolized so that it does not return to the blood 2.

It is more sensitive and specific for the estimation of GFR and less influenced by age, gender, race, muscle mass, and medication as compared to serum creatinine 6, 7, 8. Therefore, cystatin C is a promising marker of renal function. Prospective studies have shown in various scenarios that patients with increased cystatin C are at a higher risk of developing both CVDs and chronic kidney disease (CKD, 5).

However, in the recent years, cystatin C has emerged as a potential marker for cardiovascular risk. In fact, previous studies have shown that a high level of cystatin C has been related to suspected or confirmed acute coronary syndrome. The results of studies investigating the value of cystatin C in suspected stable CAD patients are controversial 9, 10, 11. But further studies have shown that high concentrations of cystatin C were independently associated with cardiovascular risk factors, such as hypertension, dyslipidemia, smoking, diabetes, and oxidative stress, in individuals without CKD or microalbuminuria 5.

Therefore, the clinical utility of cystatin C as a new predictive marker of CAD merits further investigations. The aim of this study was to investigate the role of cystatin C level as a predictor of cardiovascular events and the association between this protein, oxidative stress markers, and other biochemical risk factors for atherosclerosis in patients with CAD.

## MATERIALS AND METHODS

### Study Group

We studied 305 consecutive coronary patients (234 men and 71 women) for coronary angiography recruited from Department of Cardiology, CHU–Fattouma Bourguiba, Monastir, Tunisia. The mean age of this group is 61.15 ± 10.64 years.

All patients admitted to our study had a history of stable angina defined by the presence of chest pain or unstable angina and were found to have CAD at angiography. Two experienced cardiologists, unaware of the patients clinical history and biochemical results, visually assessed all angiograms to assess the extent of the CAD.

Patients with severe renal dysfunction, life‐threatening arrhythmias, acute and chronic liver disease, infectious and inflammatory disease, and symptomatic heart failure were excluded. The detailed histories of patients included demographics data, and cardiovascular risk factors were recorded. All participants were interviewed, and data on dyslipidemia, diabetes mellitus, hypertension, and smoking habits were recorded. For coronary risk factors, the following definitions were used: individuals were defined as hypertensive if their blood pressure was >140/90 mmHg or if they were receiving any antihypertensive treatment; individuals with a history of diabetes mellitus or those receiving any antidiabetic medication were considered to be diabetic; individuals were deemed dyslipidemic if their total cholesterol (TC) concentration was ≥5.68 mmol/l, or their triglyceride (TG) concentration was ≥2.28 mmol/l, or they were receiving lipid‐lowering drugs. Smoking history was coded as never and current smoker.

### Methods

#### Measurement of biochemical variables

Levels of TC, TG, high‐density lipoprotein cholesterol (HDLc), and serum creatinine were measured using standardized enzymatic methods (Randox‐Antrim, UK). GFR was estimated by the MDRD formula. Serum high‐sensitive C‐reactive protein (hs‐CRP), fibrinogen, and cystatin C were quantified according to the instructions of the manufacturer using particle‐enhanced immunonephelometric assay (Dade Behring, Marburg Allemagne).

#### Determination of the oxidative state markers

Superoxide dismutase (SOD), glutathione peroxidase (GR), glutathione reductase (GR), glutathione reductase (GPX) activities, and total antioxidant status (TAS) concentration were checked using commercials tests manufactured by Randox Laboratories (Antrim, UK) in a Daytona analyser. The catalase (CAT) activity was determined spectrophotometrically as the rate of substrate decomposition per unit time 12. Serum lipid peroxides were measured by the fluorimetric method of Yagi 13 using thiobarbituric acid reaction (Ex 515 nm, Em 553 nm; 14).

#### Coronary angiography

All patients underwent coronary angiography, performed using standard Judkins techniques before the start of the study. Blood samples were collected before coronary artery angiography from the antecubital vein of the patients who were resting in the supine position. The CAD severity was determined by two experienced independent observers, blinded to both the arterial waveform and clinical patient data, according to the Gensini scoring system 15 In short, the stenosis score comprises the maximum stenosis, and four CAD groups were drawn according to their CAD severity score: namely, normal coronary arteries (score 0), mild CAD (score: 1–20), moderately severe (score 20–50), and severe CAD (score >50).

### Statistical Analysis

All analyses were performed using SPSS 15.0 (SPSS Inc, Chicago, IL). Categorical variables of cystatin C groups were compared by the Chi‐square test. Continuous variables were expressed as mean ± SD and compared by one‐way analysis of covariance.

Correlations between continuous variables were assessed using Spearman's correlation analysis. A linear regression analysis was applied for CAD severity score, oxidative markers levels, homocysteine, and hs‐CRP.

In order to analyze the separate effect of CAD severity score on serum cystatin C levels and the possible interaction between the severity score and renal impairment, an analysis of covariance (ANCOVA) was used. CAD severity score and estimated GFR (eGFR) were entered as categorical independent variables, and log hs‐CRP and homocystein were entered as covariates in this analysis. A *P*‐value <0.05 was considered as statistically significant.

## RESULTS

We studied 305 patients grouped according to the CAD severity score. Cystatin C concentration was significantly elevated in the patients with a CAD severity score >50% compared to the normal coronary artery patients (2.9 ± 2.2 mg/l vs. 0.7 ± 0.2 mg/l).

The variables measured in relation with the CAD severity score are shown in Table 1. The patients with CAD severity score >1 were slightly older than those with normal arteries (*P* < 0.01). Creatinine and cystatin C were positively correlated to the increase in the CAD severity score using ANCOVA analysis (*P* < 0.01), as was the decrease in eGFR (<0.01).

**Table 1:** The Relation Between the Variables Measured and CAD Severity Score

			CAD severity score			
		0	1–20	20–50	50	*P* (ANCOVA)
Number of patients		91	93	65	56	
Gender		68H/23F	73H/20F	49H/16F	42H/14F	
Age	(years, X ± δ)	59.8 ± 9.9	60.2 ± 11.8	61.1 ± 10.1	60.5 ± 13.9	<0.01
BMI	(Kg/m^2^, X ± δ)	28.3 ± 5.1	27.6 ± 5.2	26.3 ± 3.6	24.8 ± 5.5	0.001
TC	(mmol/l, X ± δ)	4.0 ± 0.9	4.3 ± 1.4	4.3 ± 0.8	4.4 ± 0.8	<0.01
HDLc	(mmol/l, X ± δ)	1.08 ± 0.3	1.07 ± 0.4	1.07 ± 0.2	1.05 ± 0.3	<0.01
TG	(mmol/l, X ± δ)	1.8 ± 1.3	1.8 ± 0.9	1.8 ± 1.1	2.2 ± 0.6	0.03
Apo (A1)	(g/l, X ± δ)	1.5 ± 1.2	0.9 ± 0.6	0.9 ± 0.5	0.89 ± 2.2	0.001
Apo (B)	(g/l, X ± δ)	1.3 ± 0.4	1.4 ± 0.4	1.5 ± 0.6	1.7 ± 0.5	0.01
Cystatin C	(mg/l, X ± δ)	0.7 ± 0.2	0.8 ± 0.3	1.2 ± 0.9	2.9 ± 2.2	<0.01
Creatinine	(μmol/l, X ± δ)	71.1 ± 8	73.3 ± 8.01	83.2 ± 8.2	125.6 ± 6.2	<0.01
eGFR	(ml/min/1.73 m^2^, X ± δ)	96.1 ± 3.5	94.3 ± 3.2	88.3 ± 4.01	73.2 ± 5.5	0.04
Log hs‐CRP		0.62 ± 0.5	0.68 ± 0.58	0.69 ± 0.46	0.7 ± 0.56	0.01
Fibrinogen	(g/l, X ± δ)	3.9 ± 1.4	3.6 ± 1.1	4.2 ± 1.4	5.5 ± 1.7	0.01
GPX	(U/gHb, X ± δ)	62.1 ± 10.4	59 ± 10.5	56.6 ± 9.6	57 ± 10	<0.001
GR	(U/gHb, X ± δ)	2.6 ± 0.8	2.4 ± 0.8	2.29 ± 0.7	2.1 ± 0.4	0.001
CAT	(μmol H_2_O_2_/min/mg proteins)	1 ± 0.08	1.06 ± 0.04	1.02 ± 0.1	1 ± 0.05	<0.001
SOD	(U/gHb, X ± δ)	687.9 ± 454.7	662.3 ± 351.4	553.7 ± 361.3	558.8 ± 464.4	<0.001
TAS	(mmol/l, X ± δ)	1.1 ± 0.3	1.1 ± 0.2	1.10 ± 0.3	1.3 ± 0.2	<0.001
Lipid peroxides as malondialdehyde	(μmol/l, X ± δ)	1.8 ± 0.3	1.8 ± 0.3	1.9 ± 0.2	1.95 ± 0.2	<0.001
homocyteine	(μmol/l, X ± δ)	9.8 ± 5.5	12.21 ± 6.1	13.36 ± 4.3	15.91 ± 6.8	<0.001

Data are expressed as mean ± SD or number.

BMI: body mass index; H: men; F: women; TC: total cholesterol; HDLc: high‐density lipoprotein cholesterol; TG: triglycerides; Apo (A1) and Apo (B): apolipoproteins; eGFR: estimated glomerular filtration rate; hs‐CRP: high‐sensitive C‐reactive protein; TAS: total antioxidant status; GPX: glutathione peroxidase; GR: glutathione reductase; CAT: catalase; SOD: superoxide dismutase.

*P*‐value is from χ_2_ test.

The concentration of lipid peroxides measured as malondialdehyde is significantly correlated to the increase in the CAD severity score. On the other hand, our results showed a decrease in the activities of antioxidant enzyme in the patients with CAD severity score >50 (Table 1).

Subject characteristics and clinical laboratory variables of cystatin C quartile groups are presented in Table 2. The number of CAD patients increased as the quartile of cystatin C with a remarkable statistical difference between cystatin C groups (*P* < 0.001). Overall, subjects with higher serum cystatin C were older. Also, body mass index (BMI), hs‐CRP, and fibrinogen were higher at higher serum cystatin C quartile. Similarly, the concentration of lipid peroxides measured as malondialdehyde, homocytein, and CAD severity score were higher at higher serum cystatin C quartile. But HDLc and antioxidant markers (SOD, GPX, GR, CAT, and TAS) decreased progressively with higher serum cystatin C quartile. Cigarette smoking, diabetes, HTA (Arterial hypertension), and obesity are not associated with serum cystatin C.

**Table 2:** Association Between Variables of Study Population Quartiles of Cystatin C

Plasma cystatin C (mg/l)						
		Quartile 1 (≤0.615)	Quartile 2 (0.61–0.84)	Quartile 3 (0.84–1.15)	Quartile 4 (≥1.15)	*P* (ANCOVA)
Number of patients		74	76	75	80	<0.01
Gender		58H/16F	59H/17F	54H/21F	61H/19F	0.33
Age	(years, X ± δ)	52 ± 10.6	61.5 ± 10.5	65 ± 10	70 ± 10.3	<0.01
Diabetes	%	29	28	27	21	<0.01
Hypertension	%	35	34	34.9	36	<0.01
Smoking	%	14	15	14	16	0.15
Obesity	%	13	11	15	14	0.45
CAD severity score		1.2 ± 4.3	16.3 ± 14.8	20.5 ± 11.3	25.5 ± 18.3	<0.01
BMI	(Kg/m^2^, X ± δ )	25.2 ± 5	27.7 ± 5	28 ± 3	31.2 ± 5	<0.001
TC	(mmol/l, X ± δ)	3.4 ± 1.1	4.1 ± 1	4.5 ± 0.8	4.9 ± 1.1	0.031
HDLc	(mmol/l, X ± δ)	1.3 ± 0.3	0.9 ± 0.3	0.7 ± 0.1	0.6 ± 0.3	<0.05
TG	(mmol/l, X ± δ)	1.08 ± 1.1	1.4 ± 1.1	1.8 ± 1.01	2.1 ± 1.1	<0.05
ApoA1	(g/l, X ± δ)	1.3 ± 0.2	0.9 ± 0.1	0.7 ± 0.09	0.6 ± 0.1	<0.001
ApoB	(g/l, X ± δ)	1.1 ± 0.4	1.3 ± 0.4	1.5 ± 0.4	1.7 ± 0.4	<0.05
Creatinine	(μmol/l, X ± δ)	58 ± 2.8	72 ± 2.9	83 ± 3.1	90.1 ± 3.1	0.004
eGFR	(ml/min/1.73 m^2^, X ± δ)	86.2 ± 4.8	76.3 ± 3.1	72.4 ± 2.9	66.5 ± 3.01	<0.001
Log hs‐CRP		0.3 ± 0.2	0.7 ± 0.4	0.9 ± 0.2	1.01 ± 0.6	0.019
Fibrinogen	(g/l, X ± δ)	2.9 ± 1.3	3.6 ± 1.3	3.8 ± 1.1	4.7 ± 1.2	<0.001
SOD	(U/gHb, X ± δ)	822.5 ± 392	564.6 ± 392	472.8 ± 392	352.8 ± 392	<0.05
CAT	(μmol H_2_O_2_/min/mg proteins)	1.09 ± 0.1	1.05 ± 0.11	1.02 ± 0.9	1.05 ± 0.11	0.03
GPX	(U/gHb, X ± δ)	65.3 ± 10.6	60.3 ± 10.7	55.2 ± 10.2	51.2 ± 10.5	0.014
GR	(U/gHb, X ± δ)	3.1 ± 0.7	2.3 ± 0.6	2 ± 0.5	1.8 ± 0.8	<0.001
TAS	(mmol/l, X ± δ)	1.3 ± 0.22	1.1 ± 0.2	1.01 ± 0.2	0.9 ± 0.21	<0.001
Lipid peroxides as malondialdehyde	(μmol/l, X ± δ)	1.6 ± 0.3	1.9 ± 0.3	2 ± 0.1	2.1 ± 0.2	<0.05
Homocystein	(μmol/l, X ± δ)	9.8 ± 5.5	12.2 ± 5.5	14.5 ± 3.2	15.9 ± 5.5	0.03

Data are expressed as mean ± SD or number.

BMI: body mass index; TC: total cholesterol; HDLc: high‐density lipoprotein cholesterol; TG: triglycerides; Apo (A1) and Apo (B): apolipoproteins; eGFR: estimated glomerular filtration rate; hs‐CRP: high‐sensitive C‐reactive protein; TAS: total antioxidant status; GPX: glutathione peroxidase; GR: glutathione reductase; CAT: catalase; SOD: superoxide dismutase; H: men; F: women.

*P*‐value is from χ_2_ test.

Independent predictors of incident CAD, determined by logistic regression, were as follows: cystatin C, hs‐CRP, eGFR, HDLc after adjustments for age, creatinine, eGFR, homocysteine, gender, presence of hypertension, and diabetes (Table 3). Every 0.1 mg/l increase in cystatin C, 2 mg/l increase in hs‐CRP, 0.2 mmol/l decrease in HDLc, 13.7 ml/min decrease in eGFR, and 1.51 μmol/l increase in homocystein caused a 34, 12, 5, and 22% increase in the risk of having CAD, respectively.

**Table 3:** Multivariate Logistic Regression Analysis for Predicting the Presence of CAD

Variable	Odd ratio	95% CI	*P*
Cystatin C	1.215	1.139–1.281	0.001
hs‐CRP	1.123	1.117–1.130	0.012
Fibrinogen	0.989	0.952–1.028	0.022
eGFR	0.945	0.919–0.989	0.054
HDLc	0.920	0.903–0.940	0.033

CI: confidence interval; hs‐CRP: high‐density lipoprotein cholesterol; eGFR: estimated glomerular filtration rate; HDLc: high‐density lipoprotein cholesterol.

A backward stepwise multiple regression analysis was performed in the combined population of normal CAD (score 0) subjects to severe CAD patients (score >50) with cystatin C as a dependent variable (Table 4). In multivariate analysis, the CAD severity score was a significant predictor of the cystatin C concentration, as well as eGFR.

**Table 4:** Backward Stepwise Multiple Regression Analysis of Cystatin C As Dependent Variable in the Total Population

		Standard	Standard error of	
		regression	regression	
Dependent variable	Independent variables	coefficient	coefficient	Significance
Cystatine C	eGFR	−0.014	0.02	*P* < 0.01
*R* = 0.922, *P* < 0.001	Log hs‐CRP	0.173	0.013	*P* < 0.01
*F*(9.161) = 62.854	Homocystein	0.119	0.017	*P* < 0.01
	CAD severity score	0.02	0.01	*P* < 0.01

Values are expressed as number.

hs‐CRP: high‐density lipoprotein cholesterol; eGFR: estimated glomerular filtration rate.

We found that the increased level of cystatin C persisted where either hs‐CRP or homocystein levels were added as covariates in the multivariate model. Linear regression analysis showed that cystatin C, fibrinogen, hs‐CRP, TAS, and homocysteine levels were the most significant predictors of CAD severity (*r* = 0.631, *r* = 0.023, *r* = 0.37, *r* = 0.136, and *r* = 0.039; *P* < 0.05, respectively).

So, in the current study, we found that cystatin, hs‐CRP, and fibrinogen are independently correlated to the increase in CAD severity score. On the other hand, our results showed a positive correlation between cystatin C and inflammatory markers such as hs‐CRP and fibrinogen.

Our results showed a correlation between cystatin C and the levels of oxidative parameters. We observed an increase in the concentration of lipid peroxides as malondialdehyde and a decreased activity of antioxidant serum proteins associated with high concentration of cystatin C. However, cystatin C was positively correlated with hs‐CRP (*r* = 0.185, *P* = 0.02), homocyteine (*r* = 0.145, *P* = 0.036), and TAS (*r* = 0.120, *P* = 0.014). It was negatively correlated with eGFR (*r* = −0.204, *P* = 0.012), HDLc (*r* = −0.108, *P* = 0.08), and lipid peroxides as malondialdehyde (*r* = −0.204, *P* = 0.012).

## DISCUSSION

Cystatin C is a protein that belongs to a group of cysteine proteinase inhibitors, one of the four types of proteinases in mammalian cells. These types of proteins are encoded by the so‐called housekeeping genes that regulate the factors necessary for global cell function, and all nucleated cells produce them at a stable production rate 16. The protein is located extracellularly and detected mainly in biological fluids. Because of its small size, cystatin C is freely filtered by the glomerulus and is not secreted, reabsorbed, or catabolized in the proximal tubules; it does not return to the blood and is not detected in urine 17. Cystatin C has been reported to provide a more accurate and precise estimate of GFR than serum creatinine 8. In the recent years, cystatin C has emerged as a marker of cardiovascular events and mortality in different situations. For example, in patients with ischemic heart disease, cystatin C was found to be an independent risk factor together with traditional cardiovascular risk factors, renal function, or the presence of microalbuminuria 18.

The study of Koc et al. 1 assessed a cut value of cystatin C as 0.82 mg/l with a 75.5 and 75% sensitivity and specificity, respectively. However, several studies have demonstrated that CKD patients are known to be at an increased risk of developing CVD and cardiovascular events 5.

In this study, cystatin C was correlated with CAD severity score, BMI, HDLc, creatinine, homocyteine, lipid peroxides as malondialdehyde, and GPX. Thus, cystatin C appears to be a marker of cardiovascular risk, and high correlations of circulating cystatin C have been shown to be consistently and strongly associated with the occurrence and the severity of CVD. Our results agree with the previous idea that demonstrates the positive correlation between cystatin C and the increase in the CAD severity score.

Moreover, cystatin C seems to offer more complete prognostic information than other markers of renal disease. Above all, cystatin C appears to be useful for identifying individuals at a higher risk for cardiovascular events among patients classified as belonging to a low category according to both creatinine and eGFR value 5.

For secondary cardiovascular events, cystatin C is one of the strongest risk predictors. The contribution of cystatin C in a multivariate model remains significant, which indicates its added value to established risk factors. Because of the association of renal dysfunction with CVD, it is unclear whether cystatin C is a direct marker of CVD or merely a marker for renal failure, which has implications for therapeutic intervention 19.

Recently, Zethelius et al. 20 assessed whether a combination of biomarkers, including cystatin C, N‐terminal pro‐brain natriuretic pepetide, troponin, and CRP, improved patient risk stratification compared to established cardiovascular risk factors 5. So, a prospective cohort study by Luc et al. 10 showed an association between cystatin C and the incident of CAD. However, inflammation, associated with atherogenic changes, may be one mechanism associated with cystatin C and cardiovascular risk, and high concentrations have been found to be associated with high concentration of CRP 21, 22.

This suggested that the predictive value of cystatin C is not dependent, although, in another study, cystatin C, independently, predicted smooth CAD lesions but not complex lesions or CAD severity 10. Our study is in accordance with the result of Wang et al. 23, Koeing et al. 24, and Koc et al. 1, that among markers of renal function, cystatin C and eGFR are significantly and independently related to the presence of CAD and the CAD severity score.

Some studies found no relationship between cystatin C levels and CRP, and the association between cystatin C and cardiovascular mortality was independent of the CRP. Thus, our result is in agreement with some studies 25, 26 that suggested the presence of a positive correlation between cystatin C and CRP or fibrinogen.

Among all predictors evaluated, cystatin C was the strongest correlate of fibrinogen and the second strongest correlate of CRP. Although it has been suggested that high cystatin C concentrations are directly related to both inflammation and atherosclerosis 27, inflammation, associated with atherogenic changes, may be one mechanism associated with cystatin C and cardiovascular risk, and high cystatin C concentrations have been found to be associated with high concentrations of CRP 21.

Our results are in agreement with the previous idea that demonstrates the correlation between cystatin C and the levels of oxidative parameters, although various studies have demonstrated an association between oxidative stress markers and CKD and CAD events 28. Elevated homocyteine level is a well‐known cardiac risk factor for the development of atherosclerosis 29. Likewise, it is the most significant parameter in our study to predict serum cystatin C and the CAD severity score.

Previous studies have demonstrated that high concentrations of cystatin C have been also associated with a hypermethabolic status 30, 31. Given the various possible mechanisms responsible for the changes in cystatin C concentrations, it is conceivable that, depending on the clinical setting considered, increased cystatin C concentrations may variously reflect renal dysfunction, the effects of heart failure as a result of hypertension and/or fluid retention 32, or CAD associated with inflammation and atherosclerosis 33.

There is evidence that both elastolytic cysteine proteases and their inhibitors, an important one being cystatin C, are involved in the pathogenesis of atherosclerosis 34, 35. Inflammatory cytokines associated with atherosclerosis stimulate the production of lysosomal cathepsins, and increased plasma concentrations of cystatin C, a cathepsin inhibitor, may reflect, at least in part, an attempt to counterbalance a potentially damaging increased elastolytic activity. Studies have demonstrated that human cathepsins are expressed in endothelial cells, smooth muscle cells, and macrophages, and that they are involved in the progression, composition, and rupture of atherosclerotic plaques 36, 37.

Increased cystatin C is emerging as a marker of both CKD and cardiovascular risk. In patients with CAD, an increase in the plasma concentration of cystatin C indicates severity of coronary lesions. As the presence of CAD is one of the major determinants of the prognosis in patients with reduced renal function, the concentration of serum cystatin C is expected to be useful in detecting patients at risk of CAD.

## CONCLUSION

The results of our study indicate that serum cystatin C is as relevant as CRP. There is a considerable association between cystatin C and biochemical cardiovascular risk factors such as homocysteine, low HDL, oxidative stress markers, and CRP. With these results, we suggested that cystatin C could be used as a marker in clinical practice to predict the presence of severity of atherosclerosis in suspected CAD patients.

## References

[1] Koc M , Batur MK , Karaarslan O , Abali G . Clinical utility of serum cystatin C in predicting coronary artery disease. Cardiol J 2010;17(4):374–380. 20690093

[2] Schiffrin EL , Lipman ML , Mann JF . Chronic kidney disease: Effects on the cardiovascular system. Circulation 2007;116(1):85–97. 17606856 10.1161/CIRCULATIONAHA.106.678342

[3] Cockcroft DW , Gault MH . Prediction of creatinine clearance from serum creatinine. Nephron 1976;16:31–41. 1244564 10.1159/000180580

[4] Levey AS , Bosch JP , Lewis JB , Greene T , Rogers N , Roth A . A more accurate method to estimate glomerular filtration rate from serum creatinine: A new prediction equation, modification of diet in renal disease study. Group Intern Med 1999;130:461–470. 10.7326/0003-4819-130-6-199903160-00002 10075613

[5] Arpegard J , Ostergren J , De Faire U , Hansson LO , Svensson P . Cystatin C—A marker of peripheral atherosclerotic disease? Atherosclerosis 2008;199:397–401. 18187137 10.1016/j.atherosclerosis.2007.11.025

[6] Taglieri N , Koenig W , Kaski JC . Cystatin C and cardiovascular risk. Ann Biol Clin 2010;68(5):517–529. 10.1684/abc.2010.0466 20870574

[7] Weber JA , van Zanten AP . Interferences in current methods for measurements of creatinine. Clin Chem 1991;37(5):695–700. 2032322

[8] Hoek FJ , Kemperman FA , Krediet RT . A comparison between cystatin C, plasma creatinine and the Cockcroft and Gault formula for the estimation of glomerular filtration rate. Nephrol Dial Transplant 2003;18(10): 2024–2031. 13679476 10.1093/ndt/gfg349

[9] Laterza OF , Price CP , Scott MG . Cystatin C: An improved estimator of glomerular filtration rate. Clin Chem 2002;48(5):699–707. 11978596

[10] Luc G , Bard JM , Lesueur C , et al. Plasma cystatin‐C and development of coronary heart disease: The PRIME Study. Atherosclerosis 2006;185(2):375–380. 16046222 10.1016/j.atherosclerosis.2005.06.017

[11] Niccoli G , Conte M , Della Bona R , et al. Cystatin C is associated with an increased coronary atherosclerotic burden and a stable plaque phenotype in patients with ischemic heart disease and normal glomerular filtration rate. Atherosclerosis 2008;198(2):373–380. 17983622 10.1016/j.atherosclerosis.2007.09.022

[12] Aebi H . Catalase in vitro. Methods Enzymol 1984;105:121–126. 6727660 10.1016/s0076-6879(84)05016-3

[13] Yagi K . Assay of lipid peroxides in blood plasma or serum. Methods Enzymol 1984;105:328–331. 6727672 10.1016/s0076-6879(84)05042-4

[14] Davis BJ . Disc electrophoresis II, methods and application to human serum proteins. Ann N Y Acad Sci 1964;121:404–427. 14240539 10.1111/j.1749-6632.1964.tb14213.x

[15] Gensini GG . A more meaningful scoring system for determining the severity of coronary heart disease. Am J Cardiol 1983;51: 606. 6823874 10.1016/s0002-9149(83)80105-2

[16] Turk V , Bode W . The cystatins: Protein inhibitors of cysteine proteinases . FEBS Lett 1991;285:213–219. 1855589 10.1016/0014-5793(91)80804-c

[17] Coll E , Botey A , lvarez AL , et al. Serum cystatin‐C as a new marker for noninvasive estimation of glomerular filtration rate and as a marker for early renal impairment. Am J Kidney Dis 2000;36:29–34. 10873868 10.1053/ajkd.2000.8237

[18] Ix JH , Shlipack MG , Chertow GM , Whooley MA . Association of cystatin‐C with mortality, cardiovascular events, and incidente heart failure among persons with coronary heart disease: Data from the Heart and Soul Study. Circulation 2007;115:173–179. 17190862 10.1161/CIRCULATIONAHA.106.644286 PMC2771187

[19] van Holten TC , Waanders LF , de Groot PG , et al. Circulating biomarkers for predicting cardiovascular disease risk; a systematic review and comprehensive overview of meta‐analyses. PLoS One 2013 Apr 22;8(4):e62080. doi: 10.1371/journal.pone.0062080. Print 2013. 23630624 PMC3632595

[20] Zethelius B , Berglund L , Sundström J , et al. Use of multiple biomarkers to improve the prediction of death from cardiovascular causes. N Engl J Med 2008;358(20):2107–2116. 18480203 10.1056/NEJMoa0707064

[21] Knight EL , Verhave JC , Spiegelman D , et al. Factors influencing serum cystatin C levels other than renal function and the impact on renal function measurement. Kidney Int 2004;65:1416–1421. 15086483 10.1111/j.1523-1755.2004.00517.x

[22] Shlipak MG , Katz R , Cushman M , et al. Cystatin‐C and inflammatory markers in the ambulatory elderly. Am J Med 2005;118(12):1416. 10.1016/j.amjmed.2005.07.060 16378798

[23] Wang J , Sim AS , Wang XL , et al. Relations between markers of renal function, coronary risk factors and the occurrence and severity of coronary artery disease. Atherosclerosis 2008;197(2):853–859. 17826782 10.1016/j.atherosclerosis.2007.07.034

[24] Koenig W , Twardella D , Brenner H , Rothenbacher D . Plasma concentrations of cystatin C in patients with coronary heart disease and risk for secondary cardiovascular events: More than simply a marker of glomerular filtration rate. Clin Chem 2005;51: 321–327. 15563478 10.1373/clinchem.2004.041889

[25] Poggio ED , Wang X , Greene T , Van LF , Hall PM . Performance of the modification of diet in renal disease and Cockcroft‐Gault equations in the estimation of GFR in health and in chronic kidney disease. J Am Soc Nephrol 2005;16:459–466. 15615823 10.1681/ASN.2004060447

[26] Dharnidharka VR , Kwon C , Stevens G . Serum cystatin C is superior to serum creatinine as a marker of kidney function: A meta‐analysis. Am J Kidney Dis 2002;40:221–226. 12148093 10.1053/ajkd.2002.34487

[27] Leung‐Tack J , Tavera C , Gensac MC , Martinez J , Colle A . Modulation of phagocytosis‐associated respiratory burst by human cystatin C: Role of the N‐terminal tetrapeptide Lys‐Pro‐Pro‐Arg. Exp Cell Res 1990;188:16–22. 2158459 10.1016/0014-4827(90)90272-c

[28] Font R , Prats M , Gutiérrez C , et al. Is there a relationship between cystatin C and inflammatory status, oxidative stress and other cardiovascular risk factors in non‐diabetic patients with chronic kidney disease? Nefrologia 2009;29(3):228–235. 19554056 10.3265/Nefrologia.2009.29.3.5242.en.full

[29] Humphrey LL , Fu R , Rogers K , Freeman M , Helfand M . Homocysteine level and coronary heart disease incidence: A systematic review and meta‐analysis. Mayo Clin Proc 2008;83:1203–1212. 18990318 10.4065/83.11.1203

[30] Manetti L , Pardini E , Genovesi M , et al. Thyroid function differently affects serum cystatin C and creatinine concentrations. J Endocrinol Invest 2005;28(4):346–349. 15966508 10.1007/BF03347201

[31] Risch L , Herklotz R , Blumberg A , Huber AR . Effects of glucocorticoid immunosuppression on serum cystatin C concentrations in renal transplant patients. Clin Chem 2001; 47:2055–2059. 11673383

[32] Maurer MS , Burkhoff D , Fried LP , Gottdiener J , King DL , Kitzman DW . Ventricular structure and function in hypertensive participants with heart failure and a normal ejection fraction: The Cardiovascular Health Study. J Am Coll Cardiol 2007;49:972–981. 10.1016/j.jacc.2006.10.061 17336721

[33] Naruse H , Ishii J , Kawai T , et al. Cystatin C in acute heart failure without advanced renal impairment. Am J Med 2009;122(6):566–573. 19393984 10.1016/j.amjmed.2008.10.042

[34] Sukhova GK , Shi GP , Simon DI , Chapman HA , Libby P . Expression of the elastolytic cathepsins S and K in human atheroma and regulation of their production in smooth muscle cells. J Clin Invest 1998;102:576–583. 9691094 10.1172/JCI181 PMC508918

[35] Bengtsson E , To F , Hakansson K , et al. Lack of the cysteine protease inhibitor cystatin C promotes atherosclerosis in apolipoprotein E‐deficient mice. Arterioscler Thromb Vasc Biol 2005;25:2151–2156. 16051881 10.1161/01.ATV.0000179600.34086.7d

[36] Oorni K , Sneck M , Bromme D , et al. Cysteine protease cathepsin F is expressed in human atherosclerotic lesions, is secreted by cultured macrophages, and modifies low density lipoprotein particles in vitro. J Biol Chem 2004;279:34776–34784. 15184381 10.1074/jbc.M310814200

[37] Rodgers KJ , Watkins DJ , Miller AL , et al. Destabilizing role of cathepsin S in murine atherosclerotic plaques. Arterioscler Thromb Vasc Biol 2006;26:851–856. 16410454 10.1161/01.ATV.0000203526.75772.4b

